# Gelation and yielding behavior of polymer–nanoparticle hydrogels

**DOI:** 10.1002/pol.20210652

**Published:** 2021-10-22

**Authors:** Abigail K. Grosskopf, Olivia A. Saouaf, Hector Lopez Hernandez, Eric A. Appel

**Affiliations:** ^1^ Department of Chemical Engineering Stanford University Stanford California USA; ^2^ Department of Materials Science and Engineering Stanford University Stanford California USA; ^3^ Department of Pediatrics—Endocrinology Stanford University Stanford California USA; ^4^ Department of Bioengineering Stanford University Stanford California USA; ^5^ ChEM‐H Institute Stanford University Stanford California USA

**Keywords:** diffusion, hydrogel, rheology, shear‐thinning, yield‐stress

## Abstract

Polymer–nanoparticle hydrogels are a unique class of self‐assembled, shear‐thinning, yield‐stress fluids that have demonstrated potential utility in many impactful applications. Here, we present a thorough analysis of the gelation and yielding behavior of these materials with respect to the polymer and nanoparticle component stoichiometry. Through comprehensive rheological and diffusion studies, we reveal insights into the structural dynamics of the polymer nanoparticle network that identify that stoichiometry plays a key role in gelation and yielding, ultimately enabling the development of hydrogel formulations with unique shear‐thinning and yield‐stress behaviors. Access to these materials opens new doors for interesting applications in a variety of fields including tissue engineering, drug delivery, and controlled solution viscosity.

## Graphical abstract

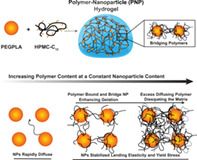

## 1 INTRODUCTION

Shear‐thinning hydrogels are unique and promising tools for controlling the delivery of therapeutics and cells, controlling solution viscosity, and 3D printing.^1^ Polymer–nanoparticle (PNP) hydrogels are a class of self‐assembled hydrogels generated from dynamic, multivalent, and entropically‐driven non‐covalent interactions between polymeric nanoparticles and high‐molecular‐weight polymers.^2^ These materials have been utilized for many applications ranging from prolonged delivery of therapeutic molecules and cells, easily applied and highly effective postoperative adhesion barriers, stabilization of biopharmaceuticals to improve cold‐chain resilience, bio‐inks for 3D printing, and prolonged delivery of wildland fire retardants for wildfire prevention.^3^, ^4^, ^5^, ^6^, ^7^, ^8^, ^9^, ^10^, ^11^, ^12^ Several important characteristics underlie why these hydrogels have demonstrated such broad utility: (i) ease of fabrication and scalability, (ii) high degree of shear‐thinning enabling facile administration by injection or spraying, (iii) rapid recovery of mechanical properties following shearing, and (iv) tunable yield stress behavior enabling them to form robust depots or coatings after application.^13^, ^14^ Additionally, when formulated at relatively high weight percent of solids (typically up to 12 wt%), these materials exhibit a small effective mesh size and thus very slow diffusion of embedded cargo compared to many other commonly used self‐assembled hydrogels (e.g., alginate).^15^, ^16^, ^17^ While recent research has revealed many of the driving factors for the dynamic mechanical properties and temperature responsiveness of materials comprising mixtures of interacting polymers and nanoparticles, many of which have focused on polymeric melt systems, the exact interaction mechanisms that dominate gelation (e.g., bridging of polymers between particles or jamming of polymer‐coated particles) under various formulation conditions are still poorly understood.^18^, ^19^, ^20^ For this reason, we sought to design and execute a series of rheology and diffusion studies to elucidate the dominant mechanisms occurring in gelation of the PNP hydrogel system.

While basic mechanical studies have been performed on these complex PNP hydrogel materials in contexts relevant for various applications, a detailed and systematic investigation on the impact of the loading of each of polymer and nanoparticle components on the resulting PNP hydrogel properties has not yet been performed. Moreover, while many rheological studies on model hydrogel systems have been performed in the past to understand mechanistic behavior, rarely are thorough mechanistic studies performed on promising novel materials, which tend to be highly structurally complex and multifaceted in their mechanical behaviors. Indeed, many studies on model materials focus on evaluating a singular material characteristic of interest, rather than the multifaceted group of material properties relevant for engineering applications. By titrating the amounts of polymer and nanoparticle in the PNP hydrogel formulation independently, it is possible to gain insight on the linear viscoelastic, yielding, and flow behavior simultaneously to further elucidate which components lead to the unique and useful properties exhibited by the PNP hydrogel system. Herein we use shear rheology and diffusion studies on a wide range of PNP hydrogel formulations to reveal the compositional features yielding the unique and desirable properties of these materials.

## 2 RESULTS

### 2.1 Formulation of PNP hydrogels

**FIGURE 1:**
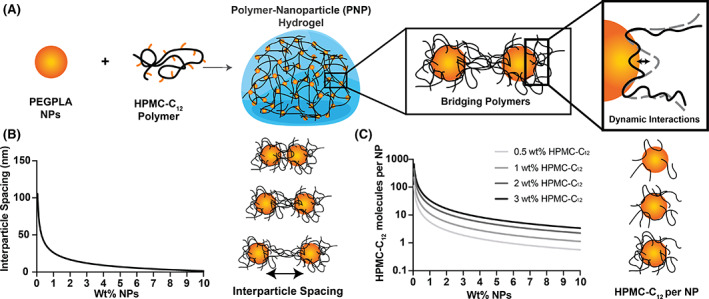
(A) PNP hydrogels form through the interactions of PEG–PLA nanoparticles (NPs) and dodecyl‐modified hydroxypropyl methylcellulose polymers (HPMC‐C_12_). Polymers bridge between polymers and dynamically interact with the NP surface. (B) Average interparticle spacing of NPs as a function of the weight percent of NPs added. (C) Number of molecules of HPMC‐C_12_ per NP as a function of the concentration of NPs and concentration of polymer

PNP hydrogels are formulated by simply mixing poly(ethylene glycol)‐*b*‐poly(lactic acid) nanoparticles (PEG–PLA NPs) and dodecyl‐modified hydroxypropyl methylcellulose polymers (HPMC‐C_12_; Figure 1A; Figures S1–S3). PEG–PLA NPs are easily scaled, biodegradable and have been used clinically in the past.^21^, ^22^ HPMC is ubiquitously used as an excipient and is easily modified.^23^ The polymers are theorized to form a dynamic corona around the nanoparticles bridging between nanoparticles.^2^, ^18^ Previous studies of PNP hydrogel systems have demonstrated that increasing the hydrophobicity of the HPMC modification, either by increasing the size of the hydrophobic moieties used for the modification (e.g., C_12_ vs. C_6_) or by increasing the total amount of hydrophobic modification along the chains, leads to stronger PNP interactions and increased hydrogel viscoelasticity,^18^ suggesting that hydrophobic interactions play a larger role in gel formation than hydrogen bonding between the HPMC and PEG polymers. Here we utilized a consistent level of dodecyl modification and concentrated our efforts on understanding the effects of P–NP stoichiometry on gelation, viscoelasticity, and flow properties. The trends observed in these studies are expected to still apply generally to PNP hydrogels if either the degree of HPMC modification or the identity of the hydrophobic moieties is adjusted.

Calculations reveal that the average interparticle spacing (IPS) of nanoparticles greatly decreases with the amount of nanoparticles added to the hydrogel (Figure 1B). Indeed, at 10 wt% NPs, the nanoparticles are nearly touching. Additionally, calculations reveal that the number of HPMC‐C_12_ polymer molecules per nanoparticle is greatly dependent on the polymer concentration (Figure 1C). Formulations are referred to in the format P–NP, whereby P refers to the weight percent of HPMC‐C_12_ and NP refers to the weight percent of the PEG–PLA NPs (n.b., the remaining mass of the formulation is phosphate‐buffered saline).

### 2.2 Network diffusion

**FIGURE 2:**
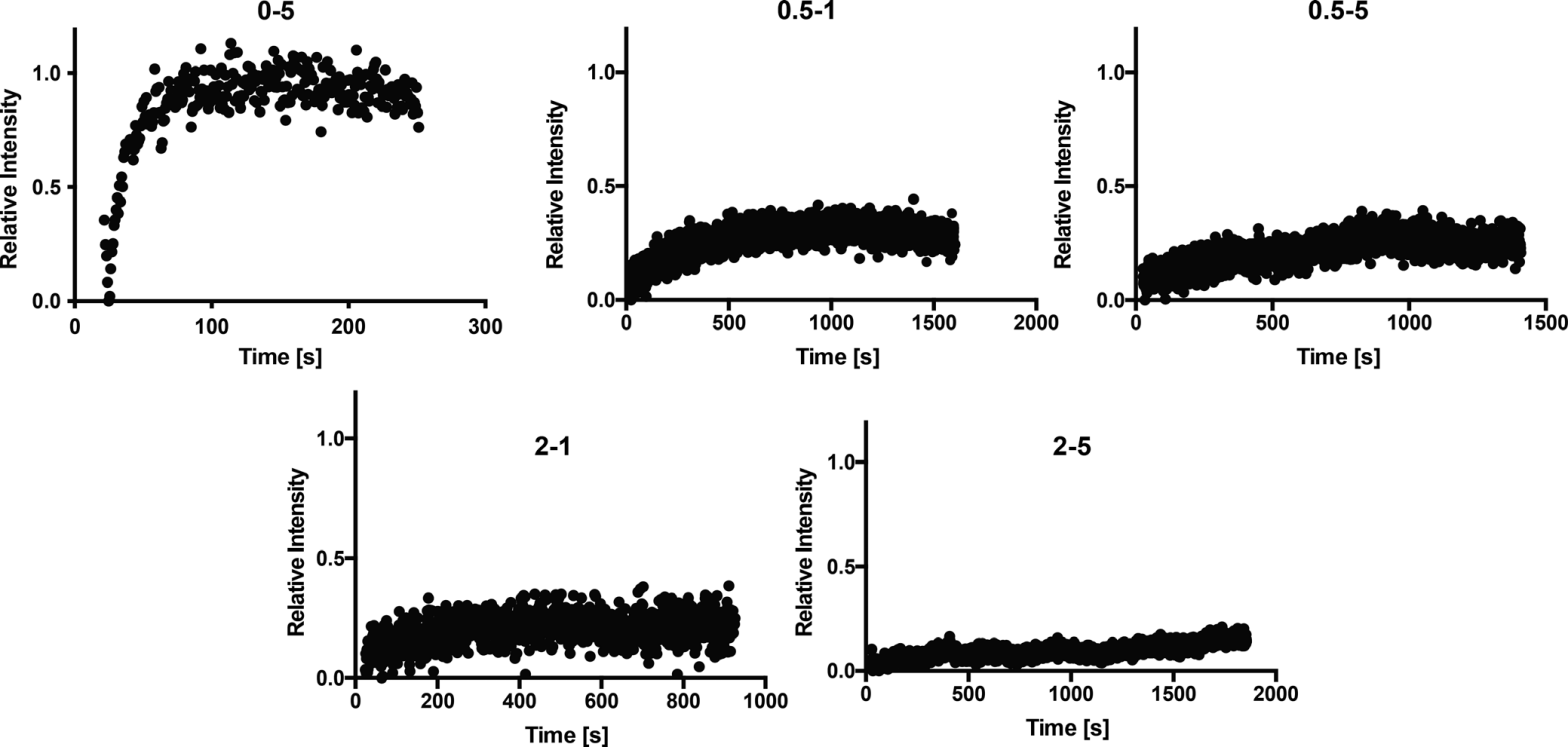
Fluorescence recovery after photobleaching (FRAP) experiments of fluorescently labeled PEG–PLA NPs were performed to evaluate NP diffusion. Recovery data are normalized by the original maximum signal and minimum signal at bleaching. Formulation notation denotes P–NP weight percent loadings in the PNP hydrogels (n.b., the remaining mass of the formulation is phosphate‐buffered saline). Representative data for each PNP formulation are shown

To gain greater insight into the structural dynamics of the components within the PNP hydrogels, fluorescence recovery after photobleaching (FRAP) microscopy experiments were performed to assess the diffusion of the polymer and nanoparticle components independently (Figure 2). The HPMC‐C_12_ and PEG–PLA NP components were first labeled with fluorescent probes to enable this analysis.^17^ A diffusion coefficient was calculated based on recovery data obtained from the FRAP experiments.^4^, ^24^ Data were collected until a plateau was reached or background photobleaching began to interfere (typically up to 30 min of data collection per FRAP experiment). An immobile fraction was calculated based on the signal that was not recovered once a plateau was reached.

We first conducted FRAP experiments with fluorescently labeled PEG–PLA NPs (Figure 2). A control solution of 5 wt% nanoparticles and no HPMC‐C_12_ polymer exhibited rapid diffusion at a rate of approximately 3.5 μm^2^/s. In contrast, the PEG–PLA NPs in hydrogels exhibited almost no diffusion and surprisingly high immobile fractions such that meaningful diffusion analysis was not feasible. These results suggest that even a very small amount of polymer crosslinks the NPs such that they become arrested in the hydrogel network, greatly reducing NP diffusion.

**FIGURE 3:**
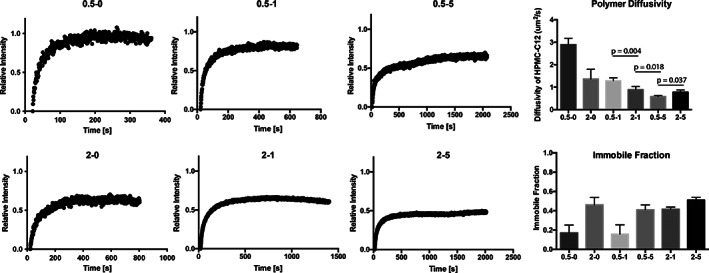
Fluorescence recovery after photobleaching (FRAP) of fluorescently labeled HPMC‐C_12_ to understand diffusion with representative recovery curves for each PNP formulation evaluated. Recovery data are normalized by the original maximum signal and minimum signal at bleaching. The diffusivity from recovery analysis of multiple samples (*n* = 3–4) and corresponding immobile fractions are presented. Formulations are referred to the polymer weight percent followed by the nanoparticle weight percent (with the rest of the weight percent being phosphate‐buffered saline)

We then sought to evaluate the diffusion of the HPMC‐C_12_ component within the PNP hydrogels (Figure 3). While polymer diffusion varied slightly with formulation, the data demonstrate that formulations with greater solid content, and thus higher viscosity, exhibited decreased polymer diffusion. Significant immobile fractions were observed in many formulations, though a comparison of the diffusion of the polymer in PNP hydrogels with a control polymer solution provides important mechanistic insight. For example, the 0.5–1 formulation exhibited much lower polymer diffusion compared to the 0.5–0 control solution, indicating that addition of only 1 wt% particles had a profound impact on polymer diffusion. In contrast, the 2–1 formulation did not have such a reduced polymer diffusion compared to the 2–0 control solution. Interestingly, even though the 2–5 formulation clearly exhibited a much higher viscosity in handling prior to running the FRAP experiments, the polymer diffusion in the 0.5–5 formulation was determined to be lower than in the 2–5 formulation.

### 2.3 PNP hydrogel gelation

**FIGURE 4:**
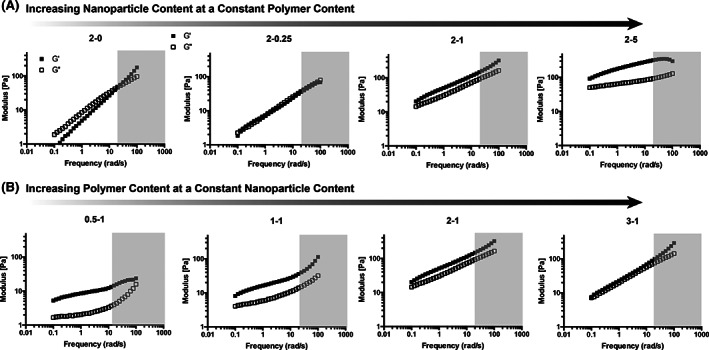
Oscillatory frequency sweeps of various PNP hydrogel formulations within the linear viscoelastic regime (1% strain). Likely inertial artifacts are shaded in gray. (A) Formulations with increasing nanoparticle content. (B) Formulations with increasing polymer content. Formulations are referred to the polymer weight percent followed by the nanoparticle weight percent (with the rest of the weight percent being phosphate‐buffered saline)

To investigate PNP hydrogel viscoelasticity, the shear rheology of formulations independently titrating the nanoparticle and polymer content was evaluated on a serrated parallel plate rheometer (Figure 4). Frequency sweeps were performed to investigate viscoelasticity across multiple timescales. Increasing nanoparticle content at a constant polymer content led to a more solid‐like rheological response. As nanoparticle content increases, the materials become stiffer and more solid‐like, exhibiting a higher G′ and lower tan (δ) (G″/G′), across a broad range of frequencies. The more shallow slope of the G′ suggests a more Rouse‐like frequency‐dependent rheological response.^25^ The crossover point of the G′ and G″ values, representing a network relaxation time, shifts from right to left with an increase in nanoparticles (e.g., shorter timescales to longer timescales), with the crossovers of the 2–1 and 2–5 formulations no longer visible in the measurable frequency range. Cole–cole representations of these data are shown in Figure S4, demonstrating that these materials do not follow Maxwell relaxation behavior, and that the addition of nanoparticles greatly alters relaxation timescales.^26^

In contrast, increasing the polymer content at a constant nanoparticle content gave a surprising result. PNP hydrogels with more polymer were generally stiffer, exhibiting a higher G′, but also demonstrated a more liquid‐like response with a higher tan (δ) and a greater frequency‐dependence of G′. Moreover, in contrast to the effect observed for addition of nanoparticles, increasing the polymer led the G′ and G″ crossover to shift to the right, indicating that increased polymer leads to shorter relaxation times. Assessing all frequency sweeps together, the results suggest that formulations with excess polymer in relation to nanoparticles (i.e., higher P–NP ratio) exhibit more dissipative characteristics similar to a polymer solution or melt. More solid content in the hydrogel, therefore, does not necessarily yield more solid‐like responses, but rather stoichiometry plays a crucial role in gelation. Similar observations have been made previously in the design of supramolecular hydrogels formed through metal–ligand interactions.^27^

### 2.4 Yielding response

**FIGURE 5:**
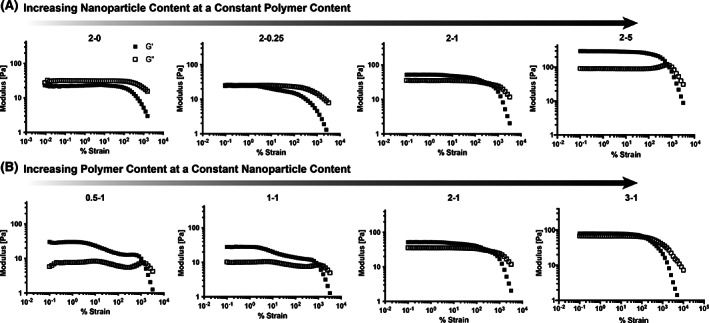
Oscillatory amplitude sweeps of various PNP hydrogel formulations at 10 rad/s. (A) Formulations with increasing nanoparticle content. (B) Formulations with increasing polymer content. Formulations are referred to the polymer weight percent followed by the nanoparticle weight percent (with the rest of the weight percent being phosphate‐buffered saline)

PNP hydrogels have shown great promise in use for biomedical research as injectable materials for controlled delivery of pharmaceuticals and cells. In order to be easily injected through standard syringes yet also form a robust depot in the body following administration, hydrogels must exhibit yield stress behavior. Additionally, injectable hydrogels, including PNP hydrogels, have been shown to reduce cell membrane damage during syringe needle injection by changing the flow velocity gradient within the syringe due to the significant shear‐thinning and yield stress flow behavior.^12^, ^28^ Thus, yielding behavior of various formulations was investigated with amplitude sweeps of strain‐dependent oscillatory rheology (Figure 5). At a constant polymer concentration, increasing the nanoparticle content led to greatly increased the strain‐to‐yield, which exceeded 500% strain for several formulations. Additionally, at a higher nanoparticle content an increase in the G″ overshoot is observed during yielding, suggesting a significant deformation energy is lost by de‐caging of the nanoparticles during the yielding response.^18^, ^19^, ^29^ In contrast, increasing the polymer content at a constant nanoparticle content exhibited the opposite effect. Increased polymer content, particularly at high polymer:nanoparticle ratios, reduced the strain‐to‐yield. Indeed, formulations with lower polymer content exhibited increases in the G″ during yielding while formulations higher polymer content did not. These results corroborate our findings described above that the stoichiometry of polymer to nanoparticle plays an important role in network dynamics.

**FIGURE 6:**
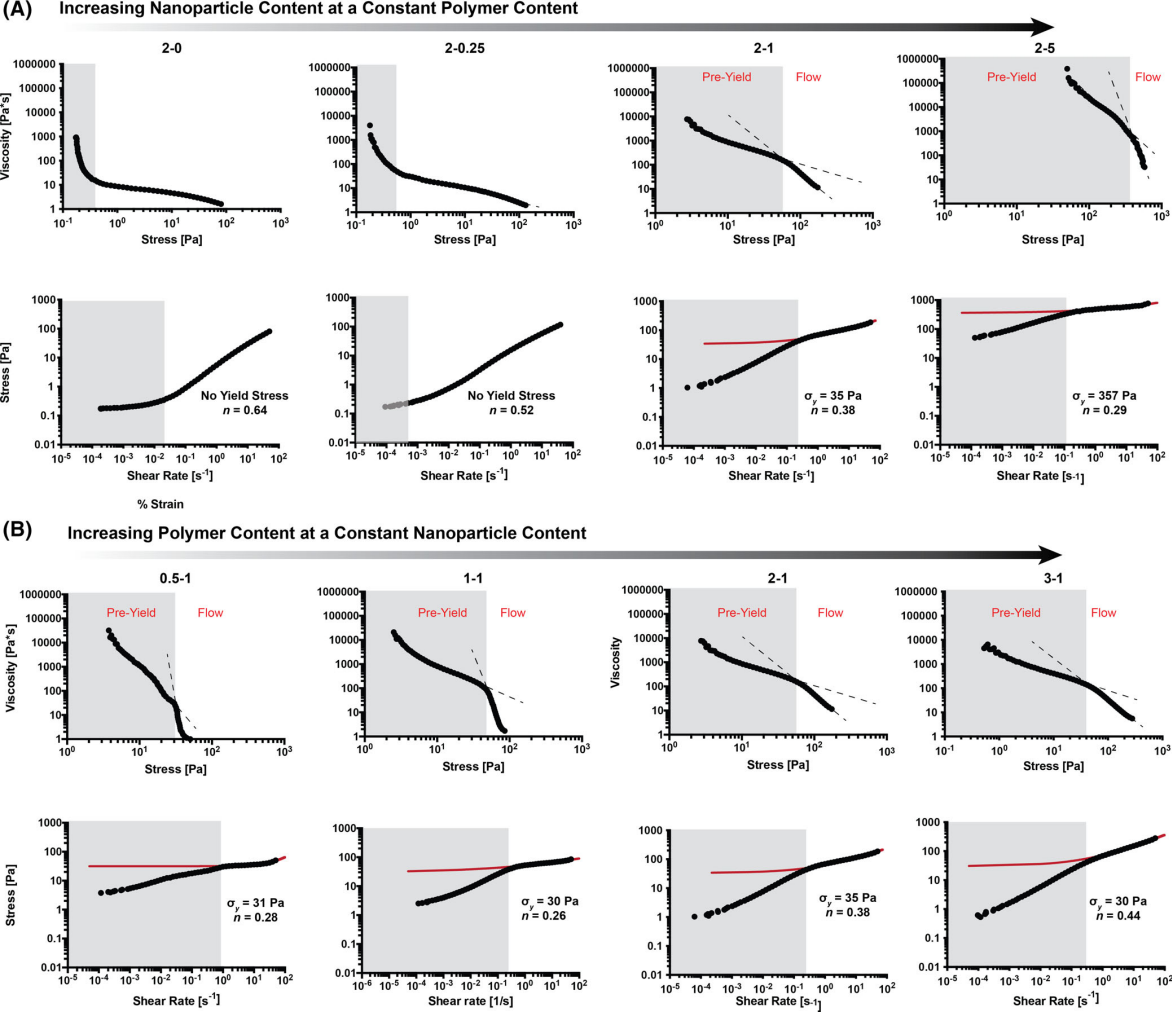
Flow sweeps of various PNP hydrogel formulations. The viscosity versus stress (top) is plotted in addition to the corresponding stress versus shear rate (bottom) for each formulation evaluated. Data that are artifact is shaded is gray. When a yield stress was observed, the pre‐yield and flow regimes are labeled. On the stress versus shear rate sweeps, yield stress values ($\sigma_{y}$) and consistency indices (*n*) from Herschel–Bulkley fits are recorded. (A) Formulations with increasing nanoparticle content. (B) Formulations with increasing polymer content. Formulations are referred to the polymer weight percent followed by the nanoparticle weight percent (with the rest of the weight percent being phosphate‐buffered saline)

In addition to examining strain, steady shear flow sweeps varying the shear rate from high to low shear rate were performed to examine the dynamic yield stress behavior of the materials (Figure 6). Measuring yield stress behavior in complex soft materials is notoriously complex.^30^ These materials exhibit unique flow‐to‐yield transitions that are best identified through visualization of the plots of the viscosity versus stress and stress versus shear rate. Upon yielding from high to low shear rate, there is a clear change in slope of the viscosity from a steep slope, characteristic of a shear‐thinning material, to a more flat slope, characteristic of a yielded material (at low enough stress and shear rate). In the yielded regime, the measured viscosity in the flow sweep is an artifact (viscosities above 1000 Pa are unbelievably highly and are almost certainly artifacts).^31^ To identify the apparent yield stress from the flow sweep, we first used tangent lines on the stress and viscosity to determine the flow regime and pre‐yield regime, and then fit the Herschel–Bulkley model to the shear‐rate dependent stress data only in the flow regime.^32^, ^33^

These studies indicate that increasing the nanoparticle content at a constant polymer content led to increased yield stresses. Indeed, measurable yield stress values were only found at nanoparticle concentrations above 0.25%. An increase in nanoparticle content also led to a lower shear‐thinning exponent from the Herschel–Bulkley fits, implying a greater degree of shear‐thinning behavior that is crucial for injectability. Interestingly, increasing polymer content at a constant nanoparticle content led to a constant yield stress across formulations, suggesting that nanoparticle content alone dictates yield stress behavior. Notably, even very weak hydrogels (i.e., low solids content and low modulus gels such as 0.5–1) exhibited significant yield stress behavior. Furthermore, while increased polymer content appeared to also increase shear‐thinning behavior, flow sweeps performed on a capillary viscometer indicated that viscosity at high shear rates is dominated by the polymer content (Figure S5), commensurate with our previous observations.^14^

### 2.5 Elucidating the design rules for PNP hydrogel formation

**FIGURE 7:**
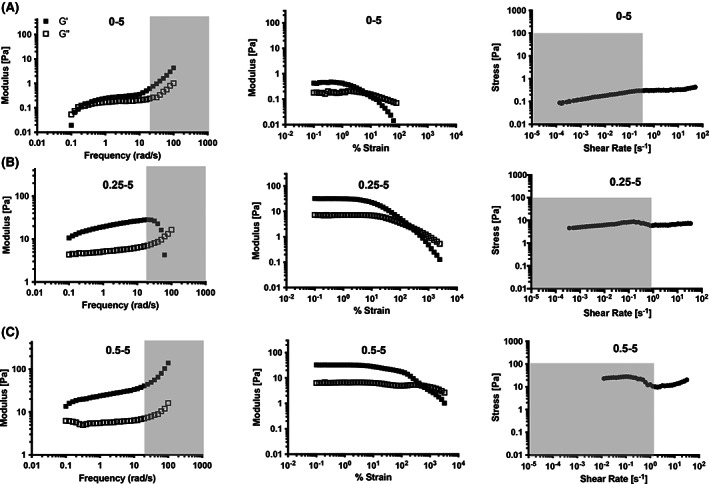
Rheological analysis of PNP formulations containing low polymer (A, 0–5, B, 0.25–5, C, 0.5–5). Formulations are referred to the polymer weight percent followed by the nanoparticle weight percent (with the rest of the weight percent being phosphate‐buffered saline)

Thus far in our studies, all formulations with at least 1 wt% nanoparticle content have been shown to exhibit robust solid‐like rheological responses. Given that in handling, nanoparticles solutions act liquid‐like, we sought to establish the point where hydrogel formation truly occurs by assessing formulations with very little polymer content but high nanoparticle content (Figure 7). While a simple 5 wt% nanoparticle solution (i.e., 0 wt% polymer content) exhibited some viscoelastic response, a crossover of the G′ and G″ was observed in the measurable frequency regime, yielding was observed at a very low strain, and the solution exhibited a very low yield stress. Upon addition of only 0.25 wt% polymer, the solid‐like hydrogel materials forms that exhibited no observable crossover of G′ and G″ in frequency sweep as well as a robust yield stress. Increasing the polymer content only slightly above this level to 0.5% yielded materials with frequency‐independent moduli, dramatically increased strain‐to‐yield, and increased yield stress values. Interestingly, the 0.5–5 formulation showed a slight increase in G″ during yielding, while the 0.25–5 formulation did not. The 0.5–5 formulation also exhibited a lower strain‐to‐yield than the 0.5–1 formulation, indicating that a higher nanoparticle content and lower polymer‐to‐nanoparticle stoichiometry increases the strain‐to‐yield. Herschel–Bulkley analysis was not possible for the flow data obtained for these formulations due to the significant artifacts in the data, leaving too few points for an effective fit.

## 3 DISCUSSION

**FIGURE 8:**
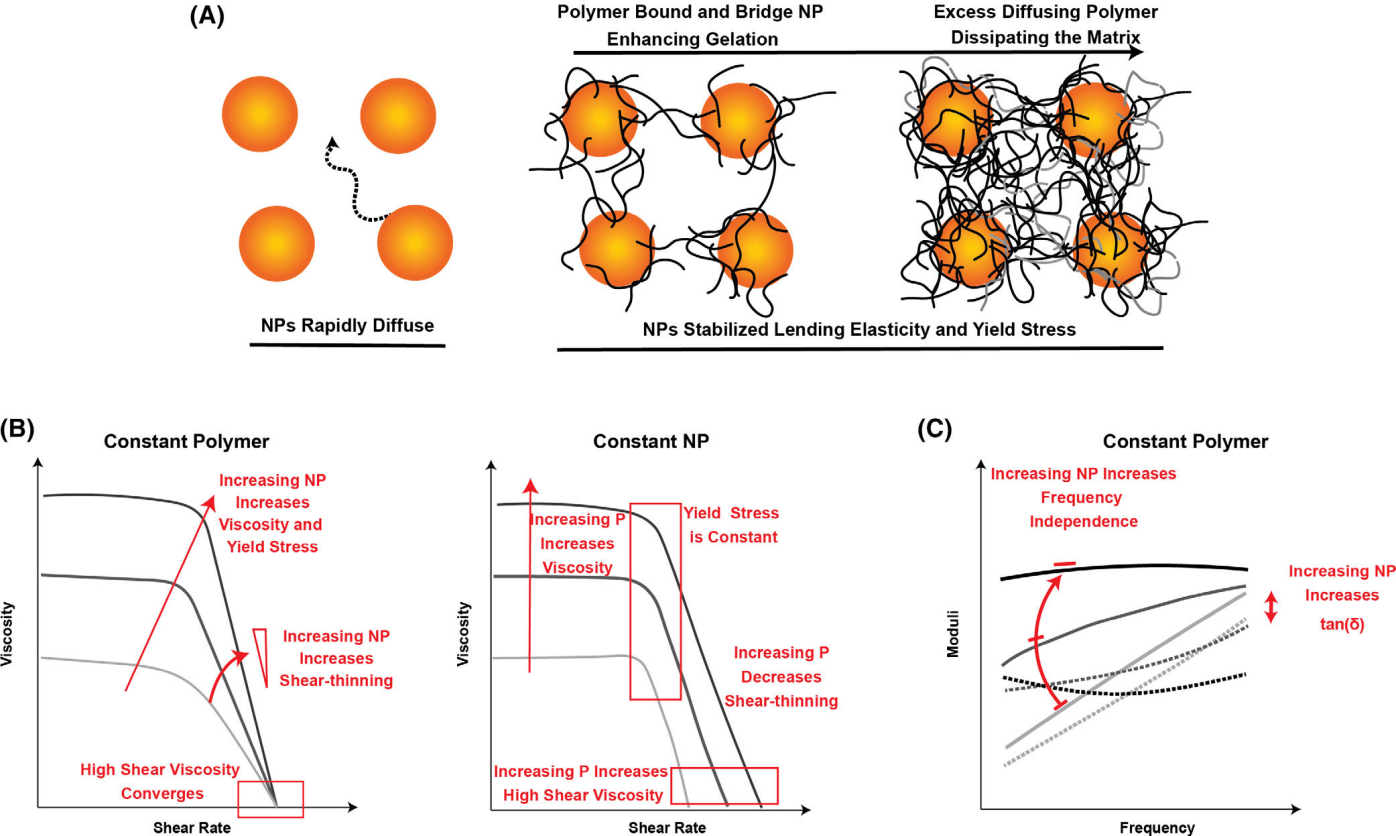
(A) Schematic representation of the structural dynamics of the PNP hydrogel with increasing polymer. (B) Schematic fictional data representations with annotation of the effect of nanoparticle increase with constant polymer content and polymer increase with constant nanoparticle content in the viscosity with increasing shear rate. (C) Schematic fictional data representation with annotation of the effect of adding nanoparticle to constant polymer content

Using a combination of studies of component diffusion and bulk rheology, we have sought to elucidate the structure and gelation mechanisms underlying PNP hydrogels. FRAP provides information on the dynamics of the individual components of the system. Through FRAP diffusion studies we discovered that the PEG–PLA nanoparticles in the hydrogel are essentially arrested into a static network structure, while the HPMC‐C_12_ polymers dynamically diffuse through this structure (Figure 8A). Increasing polymer content does not necessarily slow network dynamics, but rather too much excess polymer actually leads to “free” polymer that is not bound to nanoparticles. These free polymers therefore do not contribute to network formation and simply dissipate stress within the matrix.

A series of rheology studies corroborate these diffusion studies and demonstrate which components contribute to the unique mechanical characteristics of the PNP hydrogels. For example, the nanoparticles uniquely determine the yield stress (Figure 8B). Hydrogels with relatively higher NP content exhibit higher yield stresses, higher degrees of shear thinning, and more frequency‐independent solid‐like hydrogel behavior in the linear viscoelastic regime (Figure 8C). In contrast, hydrogels with relatively higher polymer content are more viscous, but exhibit a constant yield stress and a higher high‐shear viscosity (i.e., relatively more challenging injectability). The mechanical properties for all of the formulations evaluated are summarized in Figure S6 and can aid in formulating hydrogel materials to meet engineering specifications for any given application of interest. This study did not explore all possible formulations but simply provided design criteria, and stiffer PNP hydrogels can be formulated through the addition of a higher percentage of nanoparticles or a higher percentage of polymer. Furthermore, understanding the design principles behind these properties may enable us to create materials which push the boundaries of currently available hydrogels. One particularly interesting result from these studies is that it is possible to generate hydrogels with extremely low moduli and viscosities, yet high yield stress values.^34^

**FIGURE 9:**
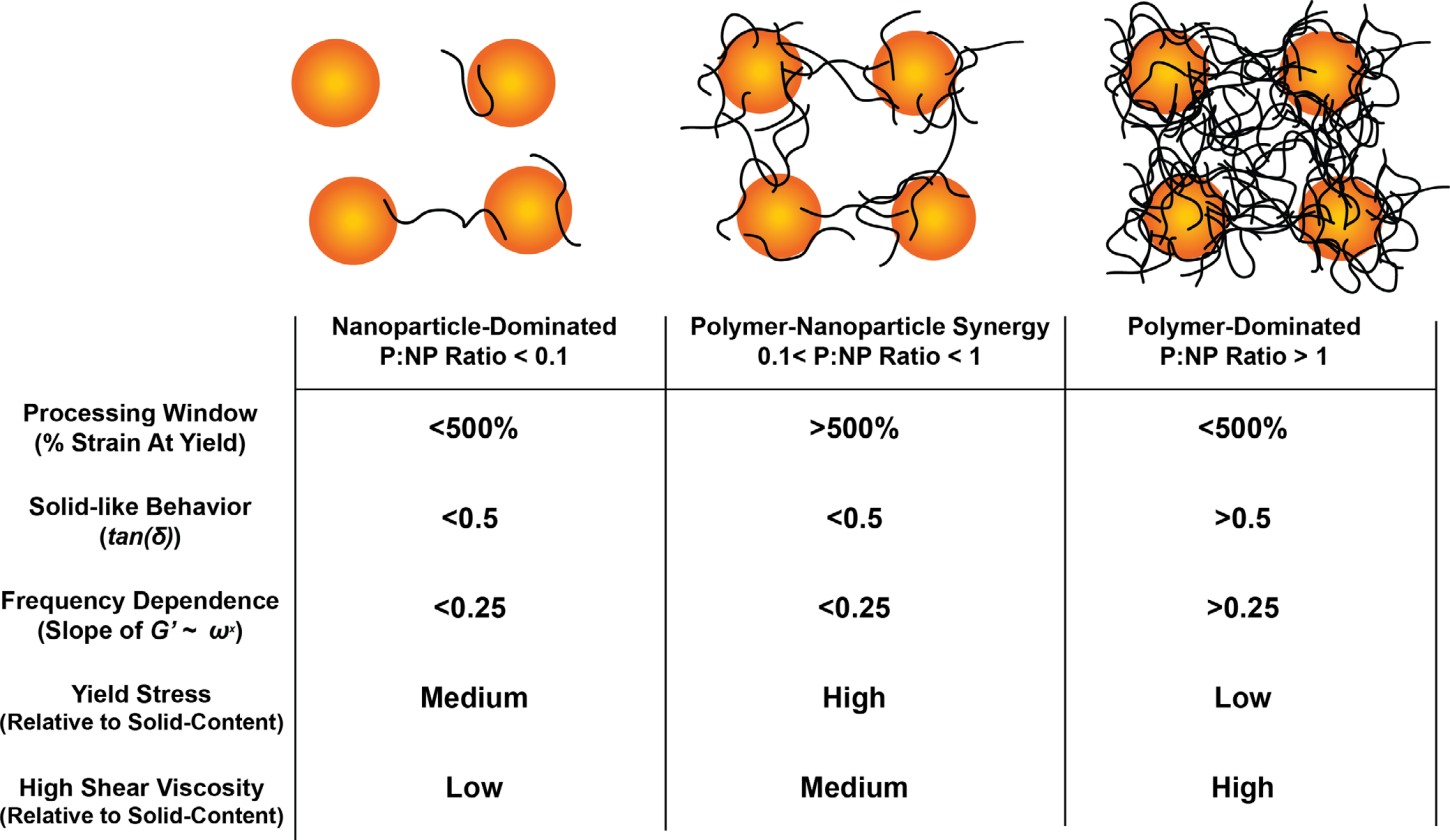
Stoichiometric regimes of PNP hydrogels based on the P:NP ratio and their resulting rheological characteristics relevant to engineering design

The results of these various studies imply that there is an optimal polymer‐to‐nanoparticle (P:NP) ratio lying between 0.1 and 1 in which there is synergy in the network leading to maximal solid‐like hydrogel mechanics and yielding behavior (Figure 9). The most synergy appeared in the 0.5–1 and 2–5 formulations, both formulations comprising P:NP ratios close to 0.5. Connecting our experimental results back to our calculations, we find that the P:NP ratio greater than 0.1 and less than 1 corresponds to a range of roughly 1–11 HPMC‐C_12_ polymer chains per PEG–PLA nanoparticle with approximately five polymers per nanoparticle being most optimal. PNP hydrogel formation seems to be independent of the nanoparticle IPS, suggesting that jamming may play a negligible role in gelation and instead bridging of polymer chains between the nanoparticles is a more dominant mechanism of gelation. From a different perspective, however, yield stress is highly dependent on the nanoparticle content, which is likely a function of the IPS. This observation corroborates literature reports that imply percolation of colloids in colloidal gels leads to yield stress behavior.^35^ Overall, thorough characterization of the PNP hydrogel platform has provided critical mechanistic insights underlying hydrogel formation and dictating hydrogel properties, enabling fine tuning of the mechanical properties for various applications of interest.

## 4 CONCLUSIONS

Our study elucidates the design features underlying the gelation and yielding behavior of PNP hydrogels. The diffusion studies conducted here indicate that the nanoparticles become nearly completely arrested into a network while the polymer dynamically diffuses through the network. We find that increasing both polymer and nanoparticle content yields stiffer materials, but that the P:NP ratio plays a large role in determining the solid‐like properties of the resulting materials. Higher P:NP ratios (i.e., excess polymer present) lead to more liquid‐like behavior, while low P:NP ratios lead to lower energy networks. We find that a P:NP ratio between 0.1 and 1 leads to synergistic effects in the network, including dominant solid‐like behavior and yielding behavior only at very high strains often in excess of 1000%. Indeed, the most optimal P:NP ratio appeared to be approximately 0.5. We additionally find that the nanoparticles play a key role in imparting a yield stress on the materials, while the polymer simply increases the viscosity at high shear rates, reducing shear‐thinning behavior. Overall, these studies reveal that manipulation of the stoichiometry and total content of the polymer and nanoparticle components in PNP hydrogels provides a means to precisely tune the mechanical properties of these materials, enabling facile optimization for various applications of interest.

## 5 METHODS

### 5.1 Materials

All chemicals, reagents, and solvents were purchased as reagent grade from Sigma‐Aldrich, Acros, or Alfa Aesar and used as received unless otherwise specified. Glassware and stir bars were oven‐dried at 180°C. When specified, solvents were degassed by three cycles of freeze, pump, and thaw. HPMC‐C_12_, and PEG–PLA were synthesized and characterized as described previously.^5^ NPs were prepared by nanoprecipitation according to literature procedures, and NP size and dispersity were characterized by dynamic light scattering (*D*_H_ ~40 nm, PDI < 0.02).^5^

### 5.2 PEG–PLA synthesis

Procedure was followed and analyzed as described previously.^5^ PEG (0.25 g, 4.1 mmol) and DBU (10.6 mg, 10 mL, 1.0 mol% relative to LA) were dissolved in dichloromethane (DCM; 1.0 mL). LA (1.0 g, 6.9 mmol) was dissolved in DCM (3.5 mL) with mild heating. The LA solution was then added rapidly to the PEG/DBU solution and was allowed to stir rapidly for 10 min. The PEG–PLA copolymer was then recovered from the reaction medium by precipitation from excess 50:50 mixture cold diethyl ether and hexanes, collected by filtration, and dried under vacuum to yield a white amorphous polymer. According to gel permeation chromatography (GPC), the resulting polymer exhibited: Mn(Đ) = 21 kDa (1.08) (full GPC trace shown in Figure S1). Mn, Mw, and dispersity values were determined via SEC implementing PEG standards (American Polymer Standards Corporation) after passing through two SEC columns (inner diameter, 7.8 mm; Mw range 200–600,000 g mol; Resolve Mixed Bed Low divinylbenzene (DVB) (Jordi Labs)) in a mobile phase of DMF with 0.1 M LiBr at 35°C and a flow rate of 1.0 mL/min (Dionex UltiMate 3000 pump, degasser, and autosampler (Thermo Fisher Scientific)).

### 5.3 HPMC‐C_12_ synthesis and characterization

Hypromellose (HPMC; 1.5 g) was dissolved in *N*‐methylpyrrolidone (NMP; 60 mL) by stirring at 80°C for 1 h. Once the polymer had completely dissolved, the solution was heated to 50°C. A solution of 1‐dodecylisocyanate (0.5 mmol, 10% dodecyl modification by weight) was dissolved in NMP (5 mL) and added to the reaction mixture followed by 150 μL of *N*,*N*‐diisopropylethylamine as a catalyst. The solution was then stirred at room temperature for 16 h. This solution was then precipitated from acetone and the HPMC‐C_12_ polymer was recovered by filtration, dialyzed within a 3.5 kDa cut‐off dialysis bag for 3 days in water, and lyophilized, yielding a white amorphous material. HPMC was found to have a molecular weight of Mn(Đ) = 272,900 (1.37) by aqueous size exclusion chromatography (SEC; full GPC trace shown in Figure S2). Aqueous SEC‐RI traces were obtained on a Optilab rEX refractive index detector (Wyatt) after passing through a column (Superose 6 Increase 10/300 GL column, Mw range of 5000–5,000,000 g/mol (GE healthcare)). 1H‐NMR was used to confirm modification (Figure S3). 1H‐NMR spectra were obtained and recorded on a Varian 600 MHz NMR spectrometer at 298 K, and chemical shifts are given in parts per million. 1H‐NMR spectra were referenced to residual proton resonances in the deuterated solvents (DMSO‐d_6_ shift = 2.50).

### 5.4 PEG–PLA nanoprecipitation

Procedure was followed and analyzed as described previously.^5^ A solution (1 mL) of PEG–PLA in acetonitrile (50 mg/mL) was added dropwise to water (10 mL) at a stir rate of 600 rpm. NPs were purified by ultracentrifugation over a filter (molecular weight cut‐off of 10 kDa; Millipore Amicon Ultra‐15) followed by resuspension in water to a final concentration of 250 mg/mL. NP size and dispersity were characterized by DLS (*D*_H_ = 35 nm, PDI = 0.02).

### 5.5 PNP hydrogel formulation

HPMC‐C_12_ was dissolved in phosphate‐buffered saline at 6 wt% and loaded into a 1 mL eppendorf tube. A 20 wt% PEG–PLA nanoparticle solution in PBS was then added to phosphate‐buffered saline and loaded into the tube. The contents were thoroughly mixed using a long spatula until gelation occurred. The tube was then spun at 10,000*g* and placed at 4°C overnight prior to testing.

### 5.6 Shear rheology

Rheological testing was performed using a 20 mm diameter serrated parallel plate at a 600 μm gap on a stress‐controlled TA Instruments DHR‐2 rheometer with a solvent trap to prevent dehydration. For lower weight percent formulations, a 40 mm plate was utilized. All experiments were performed at 25°C. Frequency sweeps were performed at a strain of 1%. Amplitude sweeps were performed at frequency of 10 rad/s. Flow sweeps were performed from high to low shear rates with steady state sensing. Duplicates for nearly all samples were performed for each test, and representative data are presented. G′ and tan (δ) values are reported at 10 rad/s, 1% strain.

### 5.7 Yield stress analysis

The yield stress was determined by first analyzing the viscosity versus stress from the flow sweep. A tangent line analysis as shown in Figure 6 was used to determine the point of yielding. Next the Herschel–Bulkley equation was fit to the stress versus shear rate data within the flow regime in Prism Software. The Herschel–Bulkley equation,

$$\sigma =\sigma_{y}+K{\dot{\gamma }}^{n},$$

where σ is the stress data, $\sigma_{y}$ K is the consistency index, $\dot{\gamma }$ is the associated shear rate, and n is the flow index was fit to determine $\sigma_{y}$, K, and n. All fits reported demonstrated $R^{2}$ values above 0.9.

### 5.8 Viscometry at high shear rates

A Rheosense m‐VROC viscometer was used to measure the hydrogel viscosity at high shear rates from low to high using a 1‐mL Hamilton syringe. Each data point was collected at steady state.

### 5.9 FRAP studies and analysis

Fluorescein isothiocyanate was coupled to HPMC‐C_12_ according to literature protocols,^4^, ^24^ and included as $\frac{1}{3}$ the wt% of total polymer added to each hydrogel. Alexa 647 was coupled to the PEG–PLA nanoparticles using copper‐free click chemistry according to previously published protocols,^36^ and included as $\frac{1}{2}$ the wt% of total nanoparticles added to each hydrogel. Gels were sealed between a glass slide and a coverslip with a 1 mm gap using an epoxy adhesive and imaged using a confocal LSM780 microscope. Samples were imaged using a low intensity laser to observe an initial level of fluorescence. Then the laser was switched to full intensity and focused on a region of interest (ROI) with a 25 μm diameter for 10 s in order to bleach a circular area. If the 10 s of bleaching did not reduce the fluorescence in the ROI by more than 15%, an additional bleach was applied. Fluorescence data were then recorded until a plateau was observed to create an exponential fluorescence recovery curve. Samples were taken from different regions of each gel (*n* = 3–4). The diffusion coefficient was calculated according to,

$$D=\frac{\gamma_{D}\omega^{2}}{4\tau_{1/2}}$$

where the constant $\gamma_{D}=\tau_{1/2}/\tau_{D}$ with $\tau_{1/2}$ being the half‐time of the recovery, $\tau_{D}$ the characteristic diffusion time, both yielded by the ZEN software, and ω the radius of the bleached ROI (12.5 μm).^24^

### 5.10 Interparticle spacing calculation

The IPS was calculated by the following equation^37^:

$$\mathrm{IPS}=2r\left({\left(\sigma_{m}/\sigma \right)}^{1/3}-1\right)$$

where $\sigma_{m}$ is 0.63, σ is the particle volume fraction, and r is the radius of the particle.

### 5.11 Statistical analysis

All error is reported as standard deviation unless otherwise specified. *P* values were calculated from Student's unpaired *t* tests.

## CONFLICT OF INTEREST

The authors declare that they have no competing interests.

## Supporting information


**Appendix S1**: Supporting information

## Data Availability

The data that support the findings of this study are available upon reasonable request from the corresponding author. The data are not publicly available due to privacy or ethical restrictions.
