# Supplementary material for: Gelation and yielding behavior of polymer–nanoparticle hydrogels
Source: J Polym Sci (2020). 2021 Oct 22;59(22):2854–66. doi: 10.1002/pol.20210652 (PMC9298381; doi:10.1002/pol.20210652)
Supplement: Supplementary file 1 — Appendix S1: Supporting information [file POLA-59-2854-s001.pdf]

Supplementary Information: Gelation and Yielding Behavior of Polymer-Nanoparticle Hydrogels

Abigail K. Grosskopf[1], Santiago Correa[2], Caitlin L. Maikawa[3], Emily C. Gale[4], Ryanne A. Brown[5], and Eric A. Appel[2,3,6,7,*]

[1]Department of Chemical Engineering, Stanford University, Stanford, CA, 94305, USA
[2]Department of Materials Science and Engineering, Stanford University, Stanford, CA, 94305, USA
[3]Department of Bioengineering, Stanford University, Stanford, CA, 94305, USA
[4]Department of Biochemistry, Stanford University, Stanford, CA, 94305, USA
[5]Department of Pathology, Stanford University School of Medicine, Stanford, CA, 94305, USA
[6]Department of Pediatrics - Endocrinology, Stanford University, Stanford, CA, 94305, USA
[7]ChEM-H Institute, Stanford University, Stanford, CA, 94305, USA

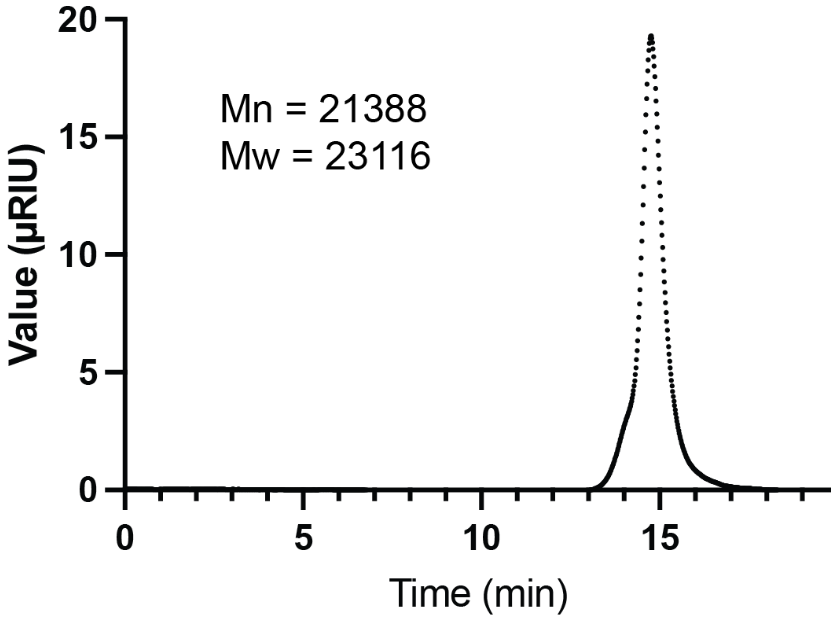

Supplementary Figure 1: Dimethylformamide gel permeation chromatography trace for molecular weight characterization of PEG-PLA polymer used for nanoparticles.

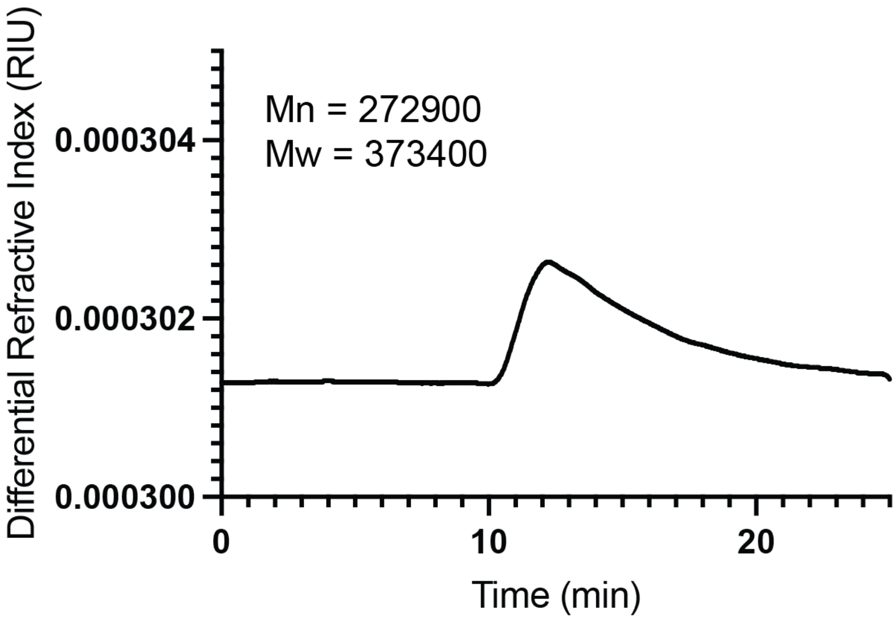

Supplementary Figure 2: Aqueous size exclusion chromatography trace for molecular weight characterization of HPMC biopolymer.

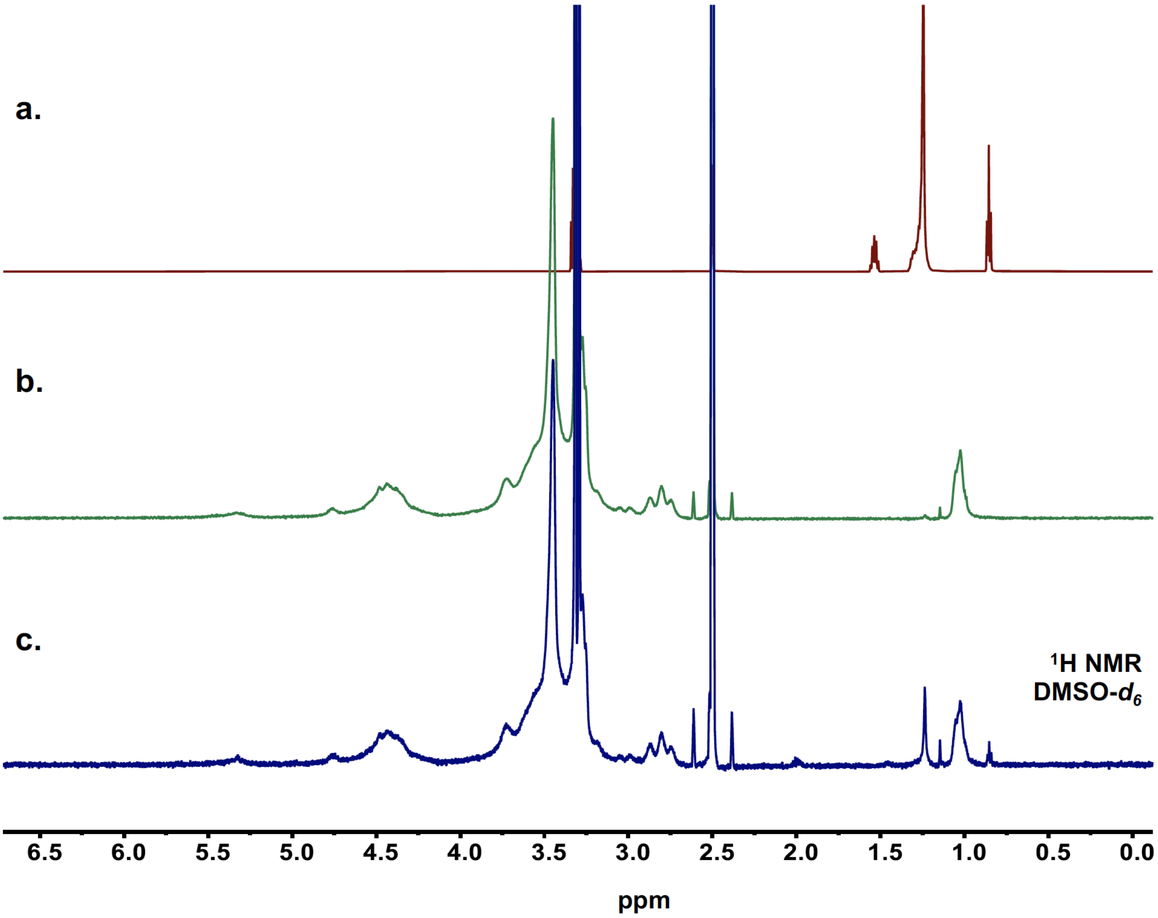

Supplementary Figure 3: a, ^{1}H-NMR (DMSO-d_6) of dodecyl/-isothiocyanate starting material. **b,** ^{1}H-NMR (DMSO-d_6) of hypromellose (HPMC) starting material. **c,** ^{1}H-NMR (DMSO-d_6) of dodecyl-modified HPMC (HPMC-C_{12}), showing the emergence of the terminal methyl group on the dodecyl side chain at 0.86 ppm, indicating the successful coupling of the dodecyl side chain to the HPMC.

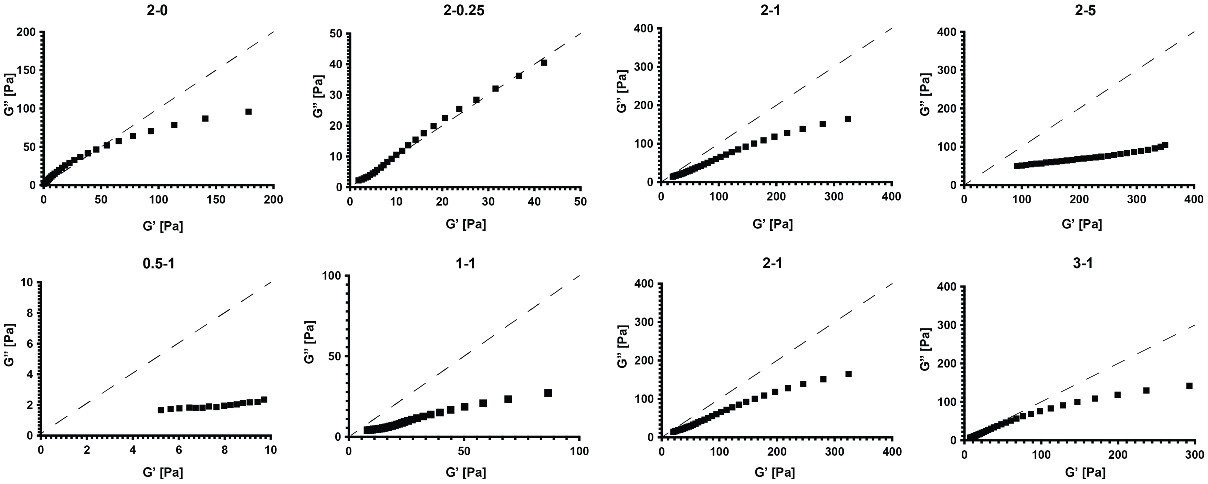

Supplementary Figure 4: Frequency sweep data for several PNP hydrogel formulations plotted in a Cole-Cole representation. The dotted line with a slope of 1 is plotted for reference. Data in the high frequency regime demonstrating inertial effects (Figure 2) is removed. Formulations are referred to in the format P-NP, whereby P refers to the weight percent of HPMC-C_{12} and NP refers to the weight percent of the PEG-PLA NPs (n.b., the remaining mass of the formulation is phosphate-buffered saline).

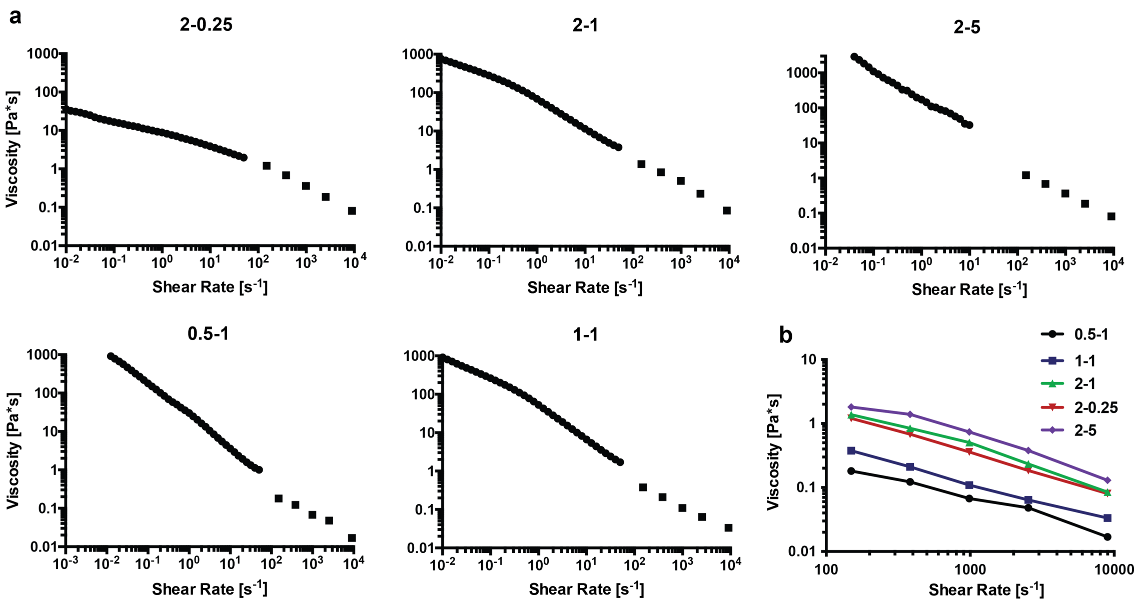

Supplementary Figure 5: a, Flow sweep demonstrating viscosity drop as a function of shear rate for PNP hydrogel formulations (last four points in each graph are from high shear rate viscometer measurements). Formulations are referred to in the format P-NP, whereby P refers to the weight percent of HPMC-C_{12} and NP refers to the weight percent of the PEG-PLA NPs (n.b., the remaining mass of the formulation is phosphate-buffered saline). **b,** Viscometry data alone of multiple PNP formulations at high shear rates.

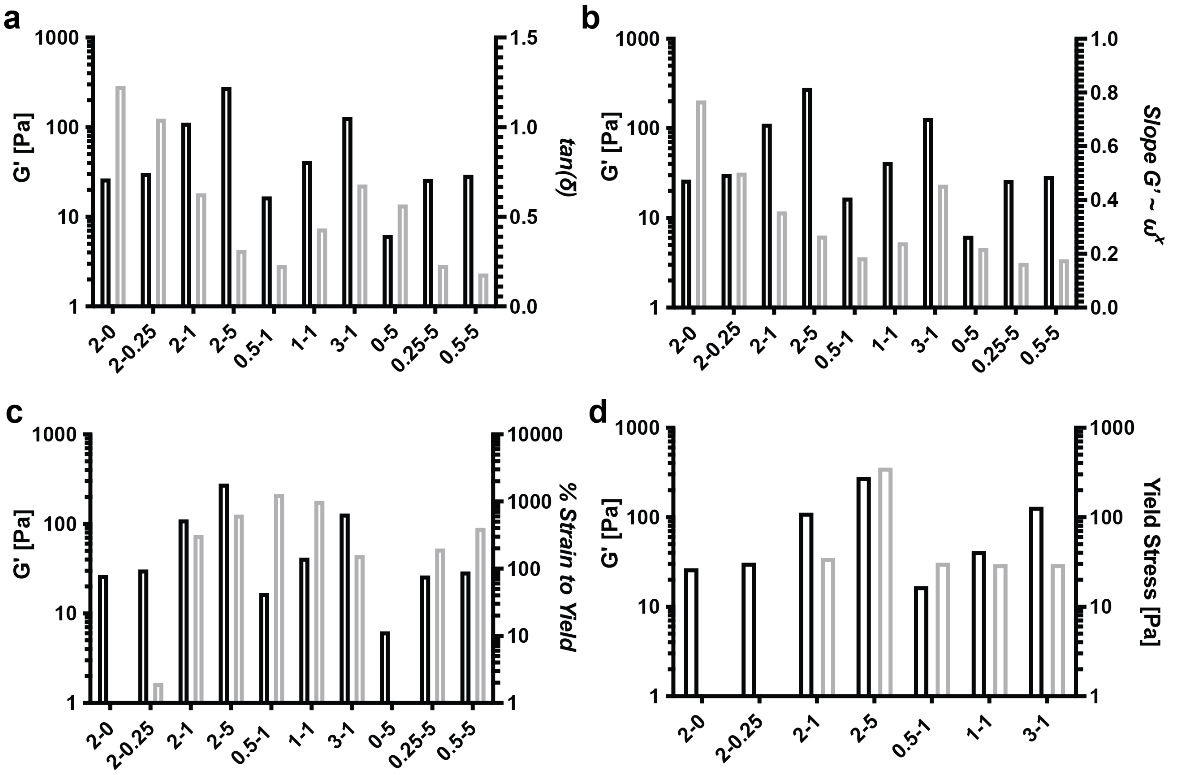

Supplementary Figure 6: **a,** Elastic storage modulus G' (black, left axis) and tan(δ) (gray, right axis) of several PNP formulations at 10 rad/s and 1% strain. **b,** Elastic storage modulus G' (black, left axis) (10 rad/s, 1% strain) and the slope of the G' across the frequency spectrum (gray, right axis) (from 0.5-20 rad/s to avoid inertial artifacts). **c,** Elastic storage modulus G' (10 rad/s, 1% strain) (black, left axis), and the % strain at yielding in the amplitude sweep (gray, right axis). The % strain at yielding is defined as the point where G' and G" intersect. **d,** Elastic storage modulus G' (10 rad/s, 1% strain) (black, left axis), and the yield stress as determined by the Herschel-Bulkley model (gray, right axis).
